# Establishment of a Novel Prognostic Prediction Model for Gastric Cancer Based on Necroptosis-Related Genes

**DOI:** 10.3389/pore.2022.1610641

**Published:** 2022-09-15

**Authors:** Zhong-zhong Zhu, Guanglin Zhang, Jianping Liu

**Affiliations:** ^1^ Department of Gastroenteroanrectal Surgery, Huangshi Central Hospital, Affiliated Hospital of Hubei Polytechnic University, Edong Healthcare Group, Huangshi, China; ^2^ Department of Abdominal and Pelvic Medical Oncology II Ward, Huangshi Central Hospital (Pu Ai Hospital), Affiliated Hospital of Hubei Polytechnic University, Edong Healthcare Group, Huangshi, China

**Keywords:** biomarker, prognosis, gastric cancer, treatment response, necroptosis

## Abstract

**Background:** Necroptosis plays a crucial role in the progression of multiple types of cancer. However, the role of necroptosis in gastric cancer (GC) remains unclear. The aim of this study is to establish a necroptosis-related prediction model, which could provide information for treatment monitoring.

**Methods:** The TCGA-STAD cohort was employed to establish a prognostic prediction signature and the GEO dataset was employed for external validation. The correlation between the risk score and the immune landscape, tumor mutational burden (TMB), microsatellite instability (MSI), as well as therapeutic responses of different therapies were analyzed.

**Results:** We constructed a prognostic model based on necroptosis-associated genes (NAGs), and its favorable predictive ability was confirmed in an external cohort. The risk score was confirmed as an independent determinant, and a nomogram was further established for prognosis. A high score implies higher tumor immune microenvironment (TIME) scores and more significant TIME cell infiltration. High-risk patients presented with lower TMB, and low-TMB patients had worse overall survival (OS). Meanwhile, Low-risk scores are characterized by MSI-high (MSI-H), lower Tumor Immune Dysfunction and Exclusion (TIDE) score, and higher immunogenicity in immunophenoscore (IPS) analysis.

**Conclusion:** The developed NAG score provides a novel and effective method for predicting the outcome of GC as well as potential targets for further research.

## Introduction

Gastric carcinoma (GC) remains a major public health problem worldwide [1]. It is the fourth most common cancer and the third frequent cancer-related mortality globally [2]. Currently, the main treatment methods for GC include a combination of surgical resection, chemotherapy, targeted therapy, and immunotherapy [3]. Due to various treatment modalities that have been developed, the 5-year survival rate for surgically treated stage IA and IB tumors is between 60% and 80%. However, the 5-year survival rate for patients with stage III tumors who undergo surgery is as low as 18%–50% [1]. Furthermore, due to the high degree of inter- and intra-tumor heterogeneity, and the fact that most diagnoses occur during advanced disease, most patients die quickly from their disease [1, 4]. Hence, it calls for innovative approaches to find critical prognostic biomarkers and elucidate the underlying mechanisms for the progression of GC.

Necroptosis is a type of programmed cell death (PCD) that differs from apoptosis and is mainly mediated by Receptor-Interacting Protein 1 (RIP1), RIP3, and MLKL [5, 6]. Existing evidence suggests that the key mediator of the necroptotic pathway can promote the metastasis and progression of cancer [5]. Some studies revealed that necroptosis is tightly associated with antitumor immunity [6]. Necrotic cancer cells attract and activate dendritic cells (DCs) that can migrate to lymph nodes and cross naive CD4+/CD8+ T cells in search of cancer antigens [7]. Meanwhile, naive T cells can differentiate into effector cytotoxic T cells and infiltrate tumors from lymph nodes and kill cancer cells. In addition, RIPK3 can also induce the expression of cytokines that activate natural killer T cells, thereby killing cancer cells [7]. Hence, necroptosis is expected to become a new target for cancer therapy. In recent years, gene expression signatures based on necroptosis-associated genes (NAGs) have been reported to predict the prognosis of several types of cancer, including colon cancer, clear cell renal carcinoma, breast cancer, and lung cancer [8–11]. However, a necroptosis-associated model in GC is lacking. In this study, we built a novel prognostic risk model based on NAGs to predict overall survival (OS) in GC patients and guide individualized treatment.

## Materials and methods

### Data Collection

We downloaded RNA-seq data as well as clinicopathological and mutation data of GC, from the TCGA database (https://portal.gdc.cancer.gov/), which included 375 GC tissues and 32 adjacent normal tissues. The TCGA cohort was used for model development, and the GSE84437 dataset that contained 433 GC samples was obtained as an external validation cohort. Patients with complete follow-up and clinical information were included in this study. The basic characteristics of the patients in the TCGA and GEO datasets are shown in Table 1. Additionally, we obtained 159 necroptosis-associated genes from previous literature (Supplementary Table S1) [12].

**TABLE 1:** Clinicopathologic characteristics of GC patients in TCGA and GEO cohorts.

Characteristics	TCGA Cohort	GSE84437 Cohort
	(*n* = 350)	(*n* = 431)
	N (%)	N (%)
Age (M±SD, years)	65.25 ± 10.33	60.02 ± 11.56
Gender		
Female	124 (35.4)	137 (31.8)
Male	226 (64.6)	294 (68.2)
Grade		
1 & 2	134 (38.3)	—
3	207 (59.1)	—
Gx	9 (2.6)	—
T stage		
T1	16 (4.6)	11 (2.6)
T2	74 (21.1)	38 (8.8)
T3	161 (46.0)	92 (21.3)
T4	95 (27.1)	290 (67.3)
Tx	4 (1.1)	—
N stage		
N0	103 (29.4)	80 (18.6)
N1-3	236 (67.4)	351 (81.4)
Nx	11 (3.1)	—
M stage		
M0	312 (89.1)	—
M1	23 (6.6)	—
Mx	15 (4.3)	—
Stage		
I	46 (13.1)	—
II	110 (31.4)	—
III	145 (41.4)	—
IV	35 (10.0)	—
Unknown	14 (4.0)	—

### Development and Validation of a Prognostic Signature Based on NAGs

To identify prognosis-related NAGs in GC, we performed univariate Cox regression analysis to screen for prognostically relevant NAGs. The Least Absolute Shrinkage and Selection Operator (LASSO) was employed to avoid overfitting and get rid of those tightly correlated genes. Subsequently, candidate NAGs were employed to construct a prognostic signature. The risk score for each patient is calculated as follows:

Risk score=regression coefficient(genei)×expression value(genei)

We stratified patients into high-risk groups and low-risk groups according to the median risk score, and the difference in OS, cancer-specific survival (CSS), and progression-free survival (PFS) between the high-risk and low-risk groups was assessed using the Kaplan-Meier (K-M) curves. Additionally, the risk score of each sample was reordered, and a risk curve and a survival status-related scatterplot were shown as a result. The area under the receiver operating characteristic (ROC) curve (AUC) was calculated to assess the accuracy of the model. Patterns of gene expression in the defined patient groups were examined via principal component analysis (PCA) with the “scatterplot3d” package in R.

The external cohort GSE84437 was employed to verify the necroptosis-related prognostic model. Stratifying patients into low- and high-risk groups were done using the same cut-off value of the training set. Then, Kaplan Meier, ROC, and PCA were performed.

### Identification of Independent Prognostic Factors and Construction of the Nomogram

We analyze the association of risk scores with clinicopathological traits, including age, gender, grade, and pathologic stage. Then, univariate and multivariate Cox regression analysis was performed to identify important predictive clinical variables for the development of genome-clinicopathologic nomogram to predict individual survival probability for GC patients. The nomogram was constructed by using R package “rms” and the accuracy of the nomogram was assessed *via* ROC and calibration curves. Moreover, we also investigated whether the risk score could affect the OS of patients in distinct clinical subgroups by log-rank test.

### Tumor Immune Microenvironment Analysis

The infiltrating score of immune cells in the TIME was calculated by Single-sample Gene Set Enrichment Analysis (ssGSEA), including 16 types of infiltrating immune cells. Moreover, the “estimate” package was used to evaluate the infiltration of immune cells and stromal cells in tumor tissues and to infer the tumor purity.

### Analysis of Therapeutic Responses and Drug Sensitivity

The tumor immune dysfunction and exclusion (TIDE) score was calculated to predict inhibitory responses to PD-1 and CTLA4 immune checkpoint in GC patients [13]. The immunophenoscore (IPS) is calculated based on the expression of various important immune molecules, including immune regulators, MHC molecules, suppressor cells, and effector cells, and can well reflect the response rate to immune checkpoint inhibitors (ICI) [17]. Moreover, the “pRRophetic” R package was employed to calculate the predicted half-maximal inhibitory concentration (IC50) of commonly applied chemotherapy drugs for obtaining the drug sensitivities of GC patients.

### Correlations of Risk Score With Mutation Status and Microsatellite Instability in GC

There is growing evidence that patients with high TMB have an acceptable response to immunotherapy and are associated with a good prognosis. We evaluated the tumor somatic mutations presented in high- and low-risk patients separately using the “maftools” R package. We further performed TMB variance analysis and correlation analysis for different risk groups. Kaplan-Meier analysis was performed to compare survival differences between low and high TMB. Subsequently, risk score and TMB were combined to perform a survival analysis to determine if there are differences in patients between different groups. We performed a combined survival analysis of risk score and TMB to explore whether the prognostic value of risk score was influenced by TMB status. Moreover, the correlation between the risk score and MSI was explored.

### Functional Enrichment Analysis

To explore the signaling pathways that necroptosis-related signatures may be involved in regulation, differentially expressed genes (DEGs) between two risk subgroups were retrieved (adjusted *p* < 0.001 and |log_2_FC| ≥ 2). Subsequently, Gene Ontology (GO) and functional annotation of Kyoto Encyclopedia of Genes and Genomes (KEGG) were performed using “clusterProfiler” R package.

### Statistical Analysis

All statistical analyses were completed by R software 4.1.0. The Spearman correlation analyses were applied to determine the correlation. Kaplan-Meier method was used for survival analysis of different risk subgroups using a two-sided log-rank test. The Wilcoxon signed-rank test was employed to compare difference of the two groups. *p* < 0.05 was the threshold of significance.

## Results

### Generation and Validation of the Prognostic Model

**FIGURE 1:**
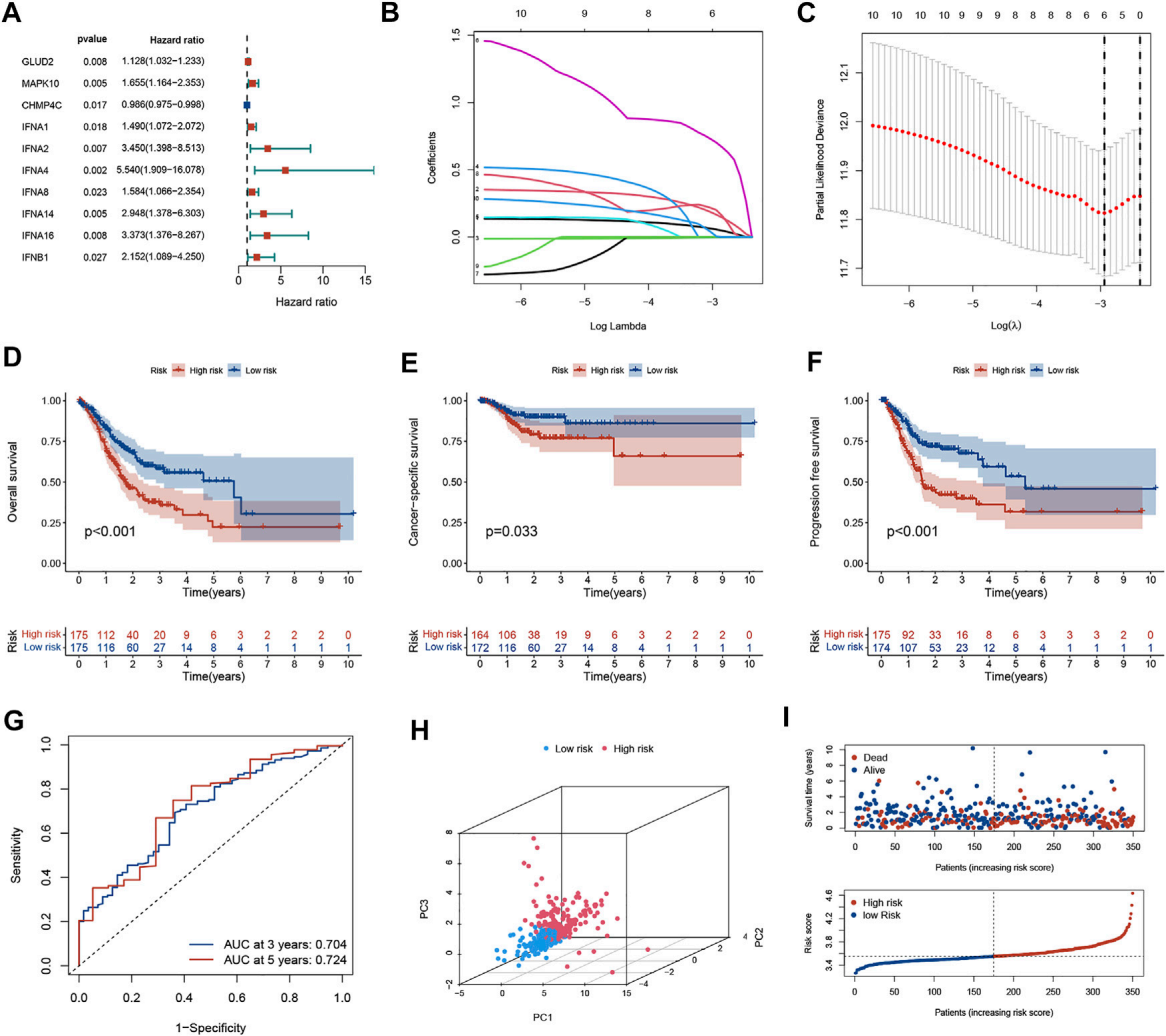
Generation of the prognostic model in the training cohort. **(A)** Univariate Cox regression analysis of 10 NAGs correlated with OS in GC patients. **(B)** LASSO regression analysis of 6 NAGs. **(C)** Cross-validation in the LASSO regression. **(D–F)** Kaplan-Meier curve indicates that the OS, CSS, and PFS of high-risk patients are significantly lower than those of low-risk patients. **(G)** The ROC curve and AUC of the model. The higher values of AUC correspond to higher predictive power. **(H)** PCA distinguishes two subgroups. **(I)** The distribution of risk scores and survival status among the two risk subgroups. NAGs: necroptosis-associated genes, GC, gastric cancer; PFS, progression-free survival; OS, overall survival; CSS, cancer-specific survival; AUC, area under the curve; ROC, receiver operating characteristic.

In univariate Cox regression analysis, a total of 10 NAGs were evidently associated with OS (Figure 1A). To minimize overfitting, cross-validation was performed in the LASSO regression (Figures 1B,C). Finally, six NAGs were employed to construct the prognostic signature. Risk score = GLUD2* 0.0562 + MAPK10*0.1662 + CHMP4C*(-0.0033) + IFNA4*0.7025 + IFNA14* 0.2007 + IFNB1*0.0054. Patients were categorized into low- and high-risk subgroups and the Kaplan-Meier plot revealed that the OS, CSS, and PFS of low-risk patients was higher than those of high-risk patients (Figures 1D–F). ROC curves presented the AUCs were 0.704 (3 years) and 0.724 (5 years), which manifested that the prognostic signature had a satisfactory predictive efficiency (Figure 1G). Besides, the PCA demonstrated that the risk score could distinguish the two risk subgroups (Figure 1H). The scatter plot risk and score distribution plot suggested that the high-risk subgroup had higher risk scores and worse survival times than that of the low-risk subgroup (Figure 1I).

**FIGURE 2:**
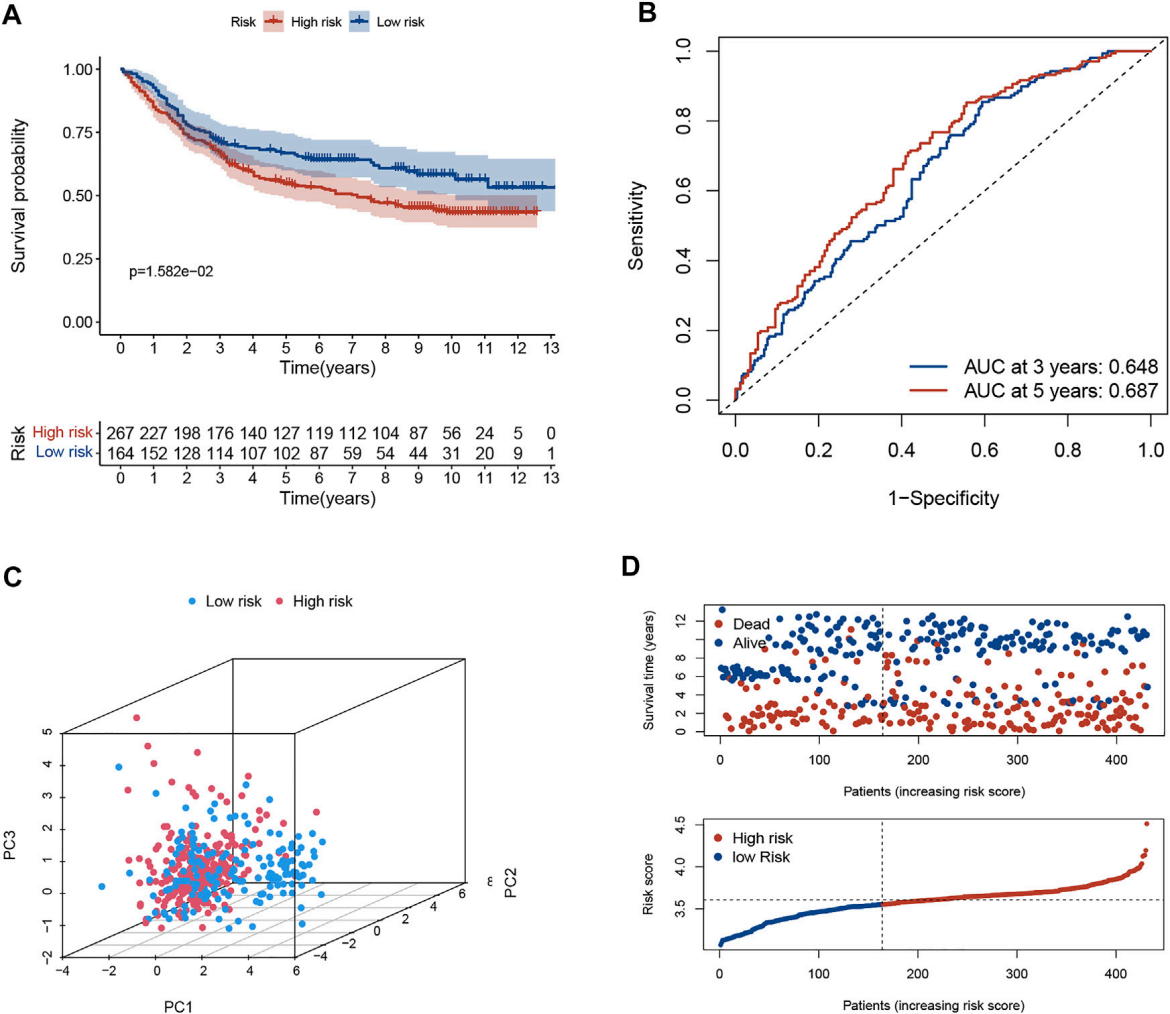
Validation of necroptosis-related signature in GSE84437 cohort. **(A)** Kaplan-Meier curves of the OS rate in the two risk groups. **(B)** The ROC curve and AUC of the model. The higher values of AUC correspond to higher predictive power. **(C)** PCA demonstrated overt separation of both subgroups. **(D)** The distribution of risk score and the coherence of survival time and survival status among two risk subgroups. OS, overall survival; ROC, receiver operating characteristic; AUC, area under the curve.

The predictive ability of the signature was well reproduced in an external validation set (GSE84437). The K-M curve revealed that patients in low-risk group had significantly favorable prognoses (*p* < 0.05, Figure 2A). The AUC value of ROC curve revealed that the risk score could predict the OS rate of GC patients to some extent (Figure 2B). PCA indicated that the patient distribution of the two risk groups was in two directions (Figure 2C). The risk curve and survival status-related scatterplot also demonstrated the same tendency of prognosis in two risk groups as the K-M curve (Figure 2D).

### Prognostic Value of the Necroptosis-Related Risk Model

**FIGURE 3:**
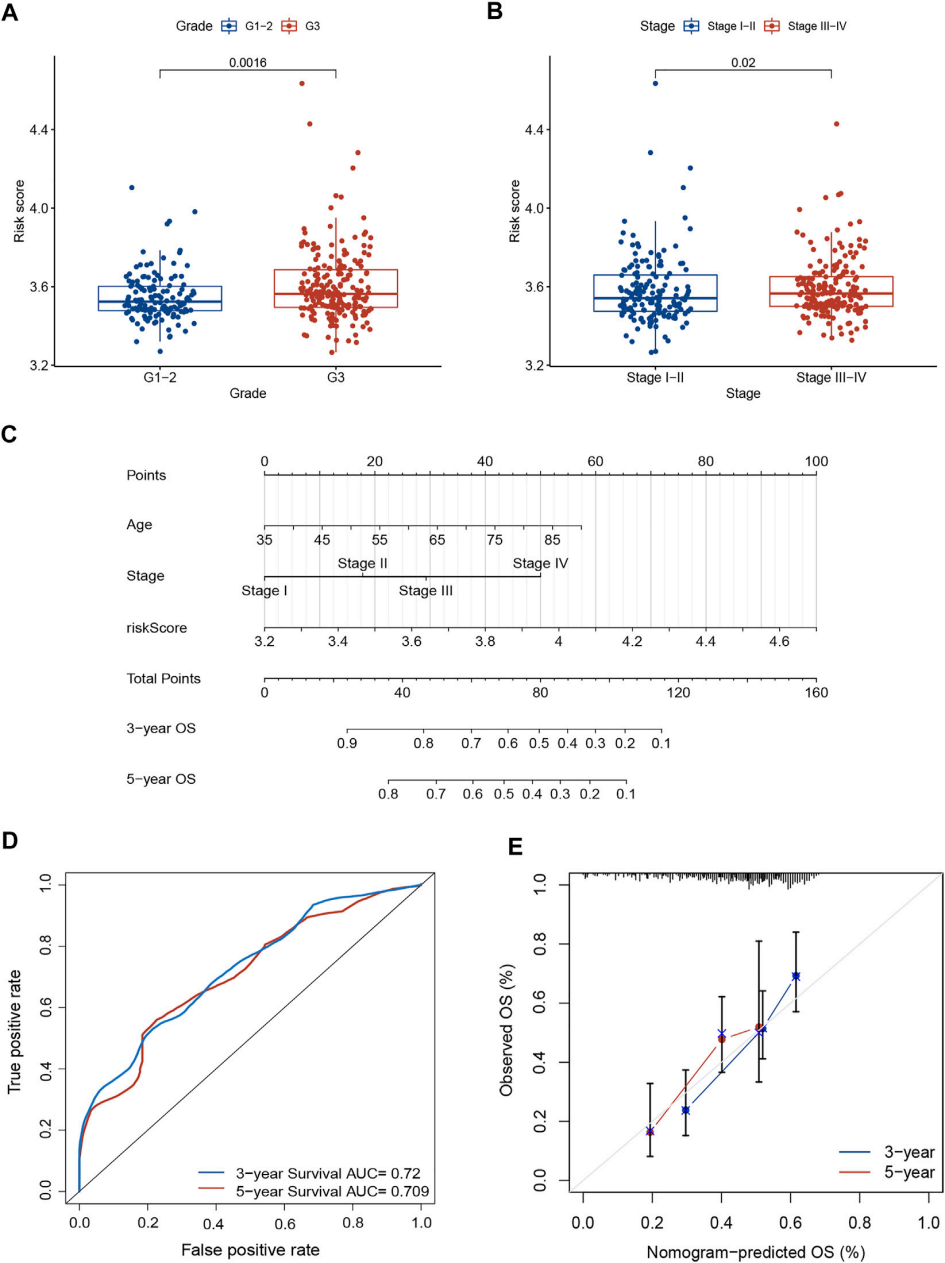
Prognostic value of the necroptosis-related risk model. **(A)** The correlation between tumor grade and risk score. **(B)** The correlation between TNM stage and risk score. **(C)** A prognostic nomogram consists of two clinicopathological variates and a risk score. **(D)** ROC curve with 3- and 5-year AUCs to assess the predictive ability of the nomogram. **(E)** The 3- and 5-year calibration plot to test the performance of the newly established nomogram. AUC, area under the curve; ROC, receiver operating characteristic.

We investigated the association between risk score and clinicopathological parameters in the TCGA training cohort. The prognostic signature observed statistically obvious higher values in G3 than in G1-2 (*p* = 0.001; Figure 3A). Similarly, a higher risk score was observed in stage III-IV than in stage I-II (*p* = 0.02; Figure 3B). To further explore the prognostic role of this signature, we performed Cox regression analyses in the TCGA cohort to select independent prognostic indicators. The results suggested that the risk score (HR: 8.137, 95% CI: 3.577–18.511, *p* < 0.001), age (HR: 1.035, 95% CI: 1.017–1.053, *p* < 0.001) and stage (HR: 1.661, 95% CI: 1.328–2.076, *p* < 0.001) were independent prognostic factors for OS (Table 2). Then, a nomogram based on three identified prognostic variables was built in the TCGA cohort to predict 3- and 5-year OS rates, respectively (Figure 3C). The ROC curves were subsequently depicted in Figure 3D, with 3-, and 5-year AUCs of 0.72 and 0.71, respectively. Furthermore, favorable consistency between prediction based on nomogram and actual observed outcomes of 3- and 5-year OS rates were illustrated in the calibration plots, respectively (Figure 3E). Additionally, we evaluated the OS of patients in two risk groups among distinct clinical subgroups, including age, gender, tumor grade, and TNM stage. As is shown in Supplementary Figure S1, patients with high risk had worse survival probabilities than those of patients with low risk in all distinct clinical subgroups.

**TABLE 2:** Results of univariate and multivariate Cox regression analyses by combining risk level with other clinical variables in the TCGA training cohort.

Group	Variables	HR	95% CI	*p*-value
Univariate	Age	1.024	1.006–1.042	0.008
Cox regression analysis	Gender (Male/Female)	1.304	0.902–1.885	0.158
	Grade (G3/G1-2)	1.337	0.955–1.872	0.091
	TNM stage (III-IV/I-II)	1.475	1.196–1.819	<0.001
	TMB	0.987	0.973–1.002	0.090
	Risk score	6.228	2.822–13.745	<0.001
Multivariate Cox regression analysis	Age	1.035	1.017–1.053	<0.001
	Gender (Male/Female)	1.221	0.842–1.771	0.292
	Grade (G3/G1-2)	1.193	0.842–1.691	0.321
	TNM stage (III-IV/I-II)	1.661	1.328–2.076	<0.001
	TMB	0.989	0.974–1.003	0.133
	Risk score	8.137	3.577–18.511	<0.001

### Tumor Microenvironment Characteristics in the High- and Low-Risk Groups

**FIGURE 4:**
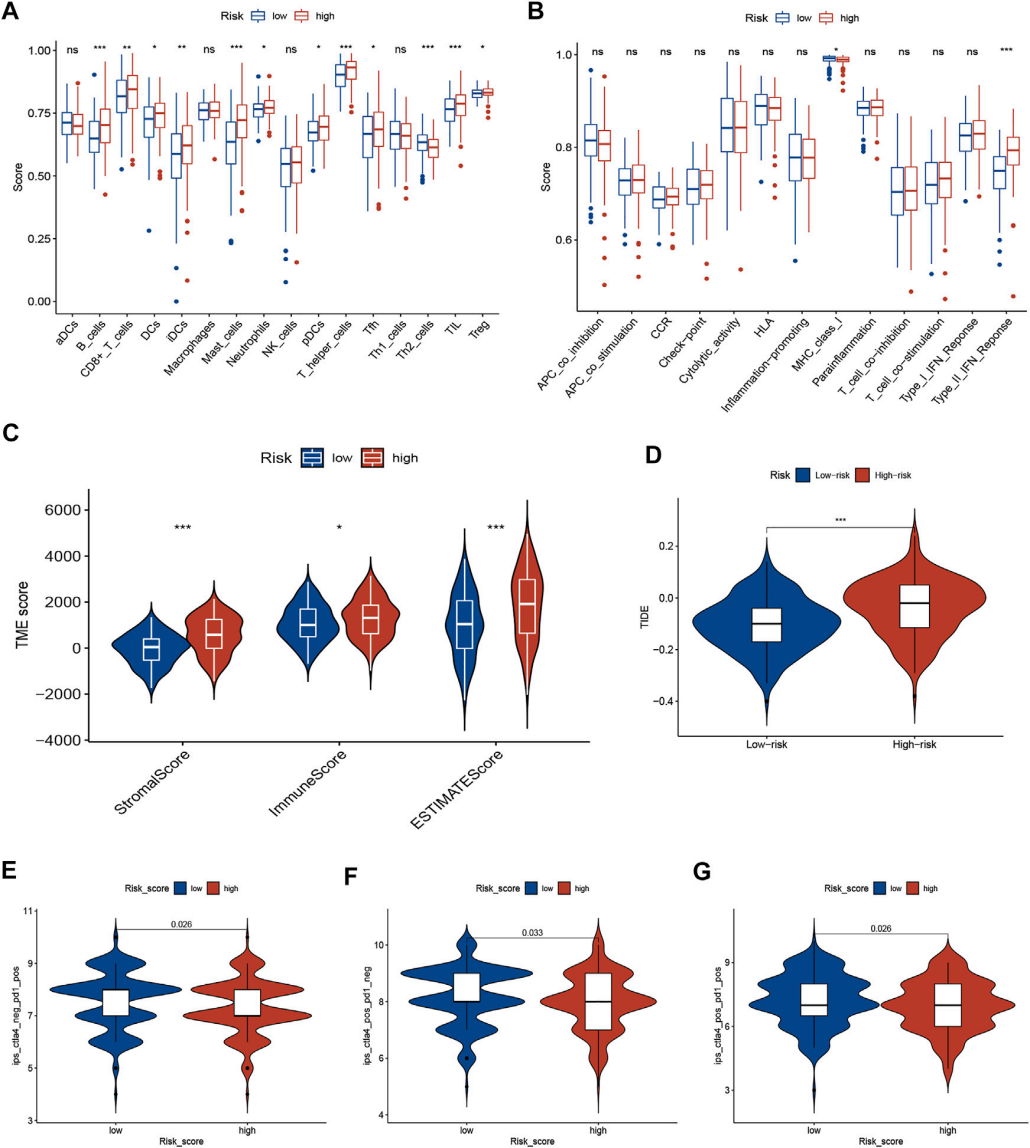
TME characteristics and ICI immunotherapy. **(A,B)** The correlation between risk score and immune cells **(A)** and immune function **(B)** was analyzed by ssGSEA. **(C)** The TIME scores between different risk subgroups. **(D)** TIDE score between different risk subgroups. **(E–G)** Immunogenicity between different risk subgroups. ICIs, immune checkpoint inhibitors; TIME, tumor immune microenvironment (TIME); Tumor Immune Dysfunction and Exclusion (TIDE).

Based on the ssGSEA algorithm, most immune infiltration cells had higher scores in the high-risk group, including CD8^+^ T cells, B cells, DCs, immature DCs (iDCs), neutrophils, plasmacytoid dendritic cells (pDCs), T helper cells, mast cells, tumor-infiltrating lymphocytes (TIL), T follicular helper cells (Tfh), and regulatory T (Treg) cells (Figure 4A). Moreover, the score of type-Ⅱ interferon (IFN) response was higher in the high-risk group, but scores of MHC-class Ⅰ were higher in the low-risk group (Figure 4B). The ESTIMATE algorithm was employed to score the TIME. The violin plot shows the ratio of TIME scores (stromal, immune, and ESTIMATE scores) between two risk groups. The TIME score of the high-risk subgroup was higher than that of the low-risk subgroup (Figure 4C).

### Necroptosis-Related Risk Model Predicts Therapeutic Benefits

The TIDE algorithm was employed to identify GC patients who can benefit from immunotherapy. The results demonstrated that TIDE scores were significantly lower in the low-risk subgroup than in the high-risk subgroup, suggesting a better response to ICI immunotherapy (Figure 4D). Furthermore, the immunogenicity of two risk groups was analyzed by IPS analysis. The ips_ctla4_pos_pd1_neg, ips_ctla4_neg_pd1_pos, and ips_ctla4_pos_pd1_pos scores were higher in the low-risk group (Figures 4E–G), suggesting that low-risk patients have a better response for immunotherapy. These results suggest that the signature can predict the response of GC patients to immunotherapy.

**FIGURE 5:**
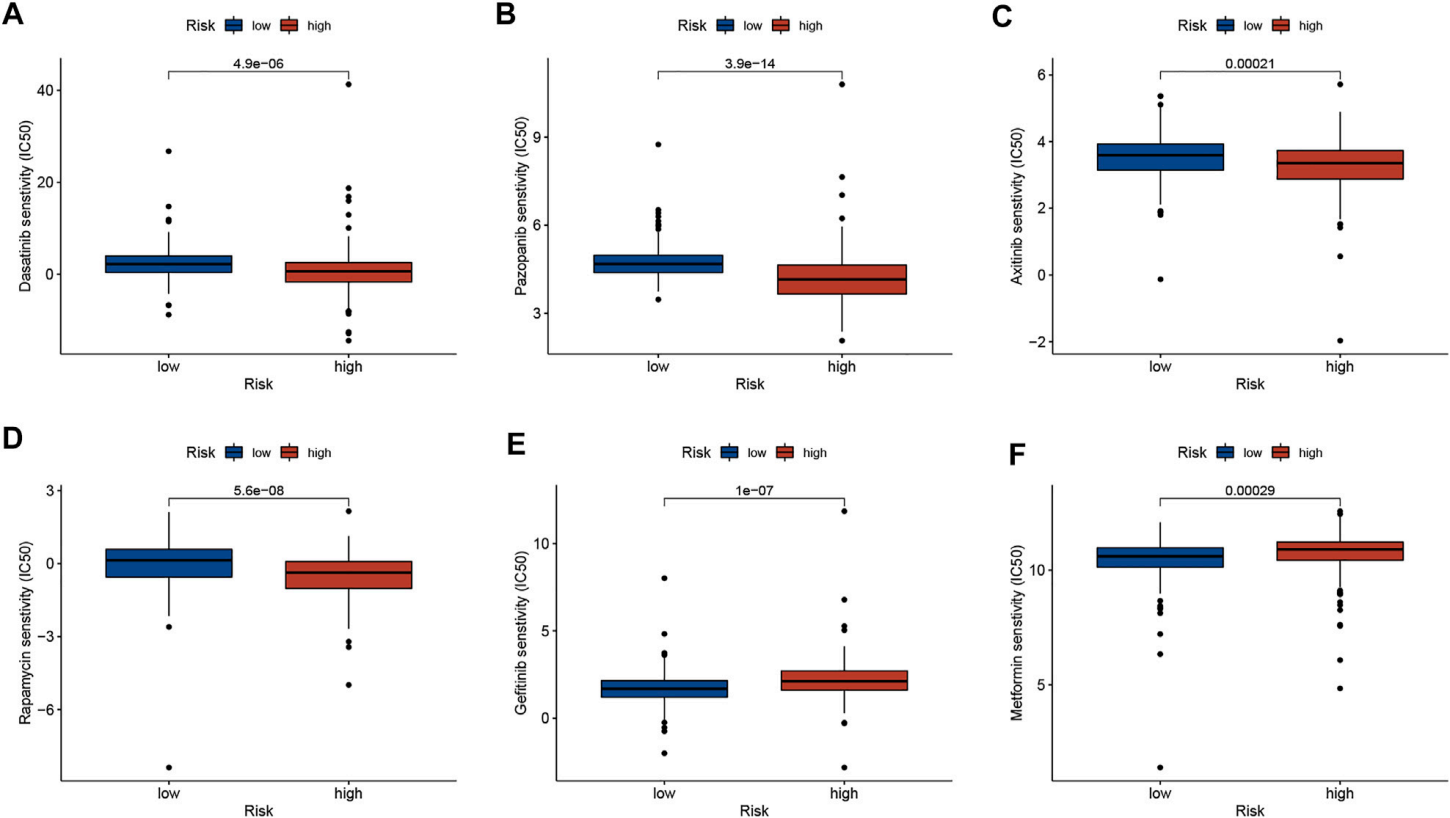
Correlation analysis between risk score and therapeutic drugs. **(A)** Dasatinib. **(B)** Pazopanib. **(C)** Axitinib. **(D)** Rapamycin. **(E)** Gefitinib. **(F)** Metformin.

To explore whether risk scores predict chemotherapy response in GC patients, we compared IC50 levels in two groups of chemotherapy drugs or inhibitors. The results are shown in Figure 5, that Dasatinib, Pazopanib, Axitinib, and Rapamycin may be potential to treat high-risk patients, while Gefitinib and Metformin may suitable for patients in the low-risk group (Figure 5).

### Correlations of Risk Score With TMB and MSI in GC

**FIGURE 6:**
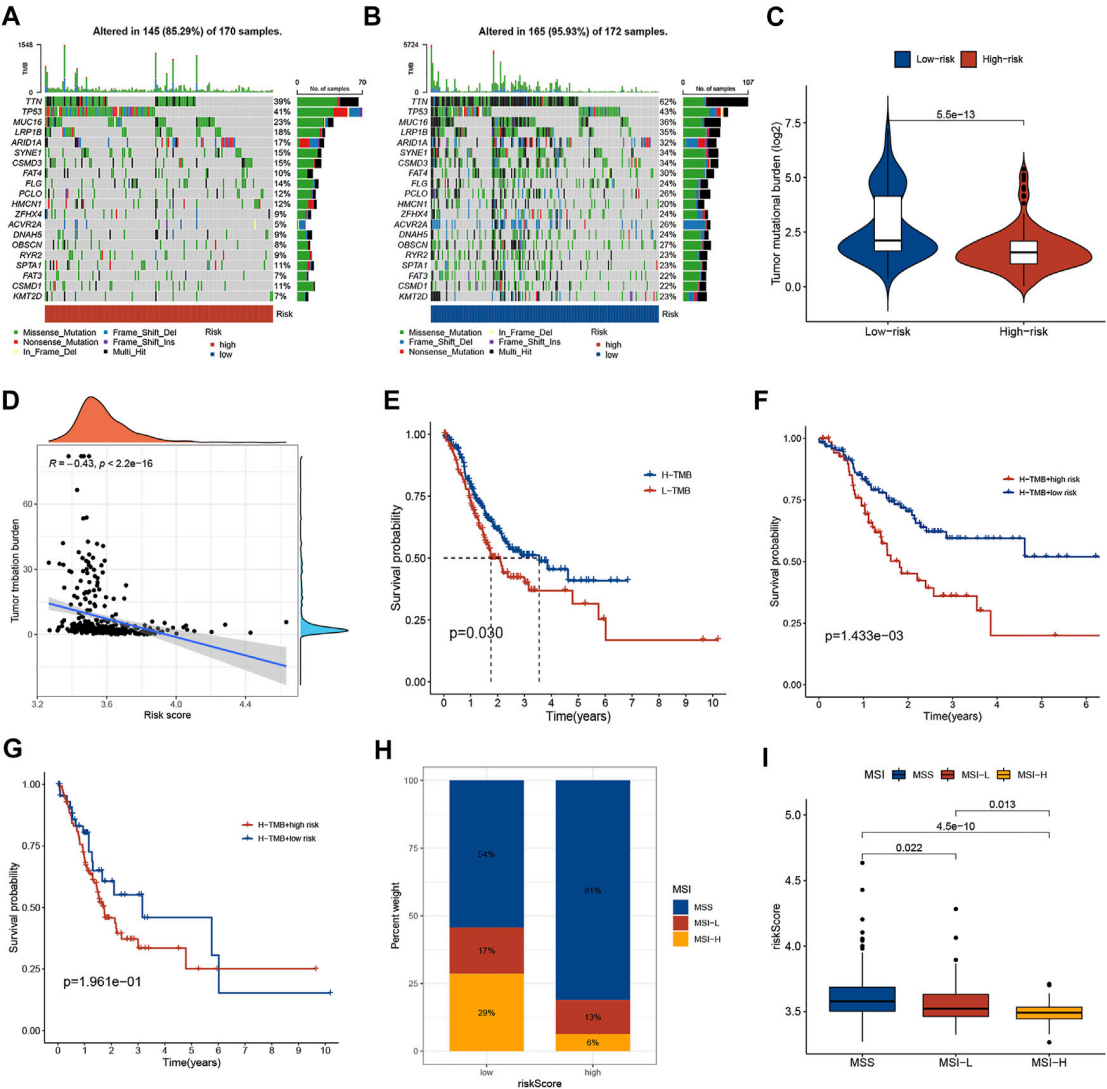
Correlations of risk score with TMB and MSI in GC. **(A,B)** The somatic frequency of GC patients in two risk subgroups. **(C)** The difference in TMB scores between different risk groups. **(D)** The relationship between risk score and TMB. **(E)** Kaplan-Meier survival curves between low and high TMB subgroups. **(F)** Kaplan-Meier survival curves of GC patients with high TMB in different risk score subgroups. **(G)** Kaplan-Meier survival curves of GC patients with low TMB in different risk score subgroups. **(H)** The proportion of different microsatellite statuses in different risk subgroups. **(I)** Risk scores between different microsatellite status subgroups. TMB, tumor mutational burden; MSI, microsatellite instability.

There is growing evidence that patients with high TMB have an acceptable response to immunotherapy and are associated with a good prognosis. A lower somatic frequency (85.29%) was frequently observed in the high-risk subgroup compared to the low-risk subgroup (95.93%) (Figures 6A,B). TMB score was obviously lower in high-risk group (*p* < 0.001; Figure 6C) and risk score was negatively correlated with the TMB score (R = −0.43, *p* < 0.001; Figure 6D). As a TMB survival curve, we found that high TMB patients have a better prognosis (*p* = 0.03; Figure 6E). A combined analysis of risk score and TMB found that the GC patients with high TMB in high-risk group had significantly shorter OS relative to low-risk patients (*p* = 0.001; Figure 6F). And the results in patients with low TMB were consistent, but not statistically different (*p* = 0.196; Figure 6G). In addition, multivariate Cox regression analysis indicated that the risk score was independent of TMB in predicting OS (Table 2). Additionally, the MSI status in the two groups was also analyzed. As shown in Figure 6H, the proportion of MSI-H and MSI-L in the low-risk subgroup was higher than that in the high-risk subgroup. Risk scores were significantly different among the different microsatellite status groups (Figure 6I).

### Functional Enrichment Analysis

**FIGURE 7:**
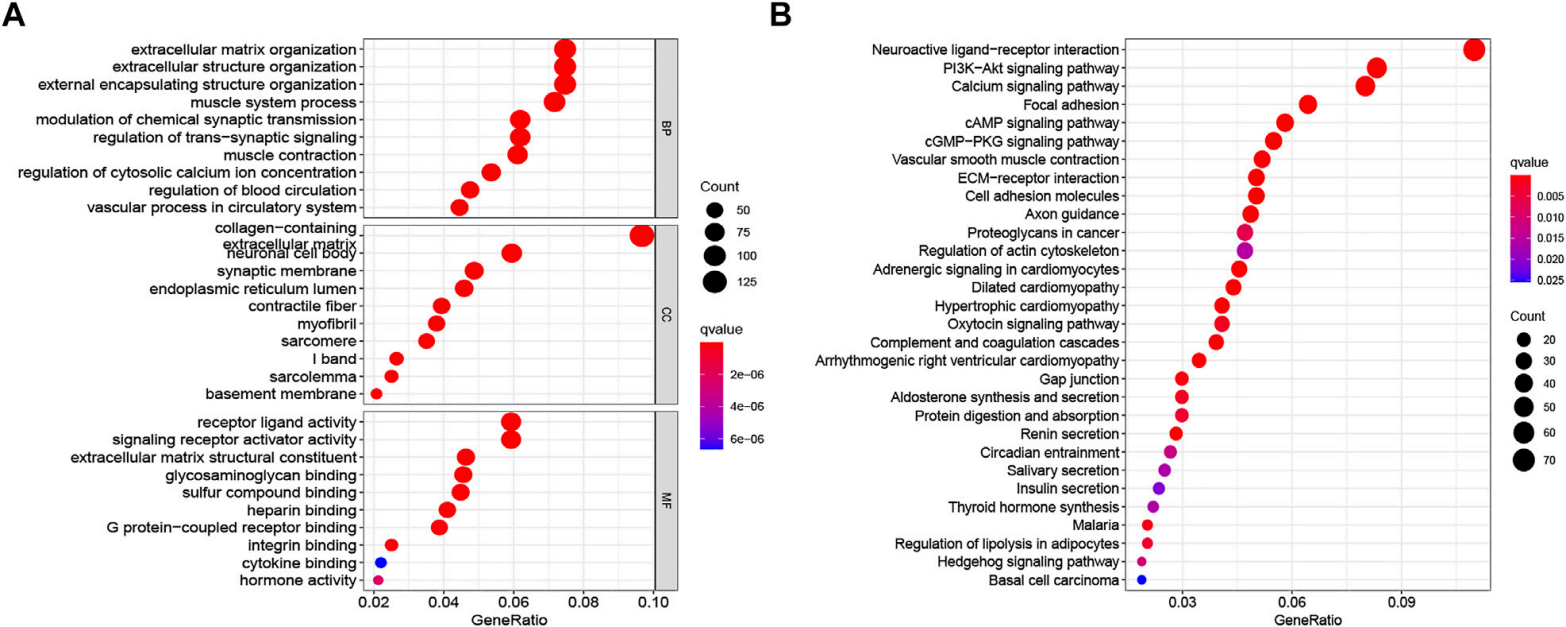
Functional enrichment analysis between low- and high-risk groups. **(A)** GO terms of DEGs among different risk subgroups. **(B)** KEGG enrichment analyses of DEGs among different risk subgroups. GO, Gene Ontology; KEGG, Kyoto Encyclopedia of Genes and Genomes; DEGs, differentially expressed genes.

KEGG pathway analyses and GO annotation were performed based on 1671 differentially expressed genes (FDR <0.001 and |logFC| > 2) between two risk subgroups. The most enriched terms in GO are extracellular matrix organization, receptor ligand activity, and collagen-containing extracellular matrix (Figure 7A); while those in KEGG with higher enrichment include PI3K-Akt signaling pathway, neuroactive ligand-receptor interaction, calcium signaling pathway, and focal adhesion (Figure 7B).

## Discussion

GC is an aggressive and heterogeneous malignancy that is a major health problem worldwide [2, 14]. The traditional prognostic evaluation system based on TNM staging can no longer meet the requirements of precision medicine. At present, reliable prognostic biomarkers for GC are lacking. In recent years, based on bioinformatics analysis, identifying biomarkers through database mining can predict the prognosis of GC [15–17]. Necroptosis is a novel model of cell death, which is caused by the binding of multiple death receptors to specific ligands by triggering specific pathogens recognition receptors such as TLR3, TLR4, and the Z-DNA sensor DAI, and induced by type I and type II interferons involving the RNA-responsive protein kinase PKR [18]. Necroptosis plays a double role in tumor regulation. On one hand, necroptosis can eliminate tumor cells and prevent tumor progression, and drug-induced necroptosis can directly inhibit tumor proliferation and metastasis [6]. In addition, necroptosis can trigger robust adaptive immune responses. On the other hand, necroptosis provides a favorable environment for tumor proliferation and metastasis [6]. Nowadays, necroptosis-related signatures have been built in several types of cancer [8, 19, 20]. Our study mainly investigated whether NAGs are correlated with the prognosis of GC and whether they could predict patient responses to immunotherapy.

In the present study, we first constructed a prognostic signature with the 6 NAGs and stratified GC patients into different subgroups. We observed shorter survival in high-risk patients compared with low-risk patients in both training and validation sets. ROC curve demonstrated that the risk score-based curve showed satisfactory prediction efficiency for training and testing cohorts. The signature contained six NAGs: MAPK10, GLUD2, CHMP4C, IFNA4, IFNA14, and IFNB1. MAPK10, a member of the MAPK family, plays a key role in cancer initiation and progression by acting as an integration point for multiple biochemical signals [21, 22]. There is increasing evidence that it acts as a microRNA target to play a tumor-promoting or tumor-suppressing role [23–25]. Gao et al. [26] revealed that miR-335-5p suppressed GC progression by targeting MAPK10. In addition, we found that IFNA4 and IFNB1 were reported in cyclic GMP-AMP synthase-stimulator of interferon (cGAS-STING) related prognostic signatures in GC [27]. GLUD2, CHMP4C, and IFNA14 have not been reported in GC.

Subsequently, multivariate Cox regression analysis indicated that the risk score was an independent prognostic indicator in GC. The prognostic role of the signature in GC was confirmed by stratified analysis. We further developed a quantitative nomogram that could evaluate the prognosis of GC patients. The clinical nomogram achieved high calibration for short-term or long-term survival prediction. Correlation analysis indicated that the risk score is positively linked to tumor grade and TNM stage. These findings indicate that the signature may be effective in determining prognosis, thereby facilitating the implementation and evaluation of the model in future clinical practice.

Recently, tumor-associated immune cells have attracted much attention. Necroptosis is an alternative mode of PCD to overcome apoptosis resistance and may trigger and amplify antitumor immunity in cancer therapy [6]. Therefore, the regulation of tumor necroptosis in the TME cell infiltration could be an important new target for GC. From the immune cell and stromal cell fractions, we can predict that immune cells may play a dominant role in the effect of TIME. We study the immune status of different groups by ssGSEA, which reveals most of the immune infiltration cells had higher scores in the high-risk group. CD8^+^ T cell infiltration generally correlated with favorable prognosis in most solid tumors [28]. Studies that examined an association between CD8^+^T and prognosis in GC are inconsistent, most studies showed a positive correlation between CD8+T and prognosis [29–31]. On the contrary, a previous study revealed that the infiltration of CD8^+^ T cells was correlated with a poorer OS and PFS in GC [32], which is consistent with our results. These findings indicate the controversial prognostic effect and heterogeneous characteristics of CD8+T cells in GC.

In recent years, immunotherapy has become a new promising approach for treating GC, especially ICIs, which have become an effective treatment [33–35]. We explored the immunotherapy benefit in GC by TIDE algorithm [13]. Patients with higher TIDE scores had a worse response to immunotherapy due to immune escape. Our results showed lower TIDE scores in the low-risk subgroup, suggesting that patients are more likely to benefit from ICI therapy. Furthermore, IPS analysis indicated that low-risk patients exhibited higher immunogenicity, implying a better response to ICI therapy. TMB was confirmed as an important determinant of ICIs efficacy and prognosis in cancer patients [36]. Our findings show a significant negative correlation between risk score and TMB and patients with the low risk had higher TMB and favorable OS. Moreover, the risk score was independent of TMB in predicting OS. Additionally, we observed an inverse correlation between risk scores and MSI status in GC patients. Taken together, the necroptosis-related signature was correlated with TMB and MSI status and has the potential to be used to predict response to immunotherapy and targeted therapy.

Nevertheless, there are still several issues to be addressed. First, clinical tissues should be used for detecting the expression level of model genes, and more functional *in vitro* or *vivo* in assays are further needed to validate their roles in the future. Second, whether the induction of necroptosis can improve the effect of immunotherapy in GC patients need urgently to be studied through clinical trials. Third, since there was a lack of a suitable externally validated cohort, the effectiveness, and reliability of the nomogram were not assessed.

## Conclusion

The developed necroptosis-related signature may serve as a predictor of prognosis and immunotherapy for GC in the future.

## Data Availability

Publicly available datasets were analyzed in this study. This data can be found here: The public datasets were obtained from TCGA (https://portal.gdc.cancer.gov/) and GEO (https://www.ncbi.nlm.nih.gov/geo/).

## Abbreviations

CSS, cancer-specific survival; GC, gastric cancer; ICIs, immune checkpoint inhibitors; IPS, immunophenoscore; LASSO, least absolute shrinkage and selection operator; MSI, microsatellite instability; NAGs, necroptosis-associated genes; OS, overall survival; PCA, principal component analysis; PFS, progression-free survival; ROC, receiver operating characteristic; ssGSEA, single-sample gene-set enrichment analysis; TMB, mutational burden.
