# Supplementary material for: Establishment of a Novel Prognostic Prediction Model for Gastric Cancer Based on Necroptosis-Related Genes
Source: Pathol Oncol Res. 2022 Sep 15;28:1610641. doi: 10.3389/pore.2022.1610641 (PMC9519854; doi:10.3389/pore.2022.1610641)
Supplement: Supplementary file 1 [file Table1.docx]

Supplementary Table 1 Summary of 159 necroptosis-related genes

| **Genes** | **Gene description** |
| --- | --- |
| TNF | tumor necrosis factor |
| TNFRSF1A | TNF receptor superfamily member 1A |
| TRADD | TNFRSF1A associated via death domain |
| TRAF2 | TNF receptor associated factor 2 |
| TRAF5 | TNF receptor associated factor 5 |
| RIPK1 | receptor interacting serine/threonine kinase 1 |
| BIRC2 | baculoviral IAP repeat containing 2 |
| BIRC3 | baculoviral IAP repeat containing 3 |
| XIAP | X-linked inhibitor of apoptosis |
| RBCK1 | RANBP2-type and C3HC4-type zinc finger containing 1 |
| RNF31 | ring finger protein 31 |
| SHARPIN | SHANK associated RH domain interactor |
| SPATA2L | spermatogenesis associated 2 like |
| SPATA2 | spermatogenesis associated 2 |
| CYLD | CYLD lysine 63 deubiquitinase |
| FADD | Fas associated via death domain |
| CASP8 | caspase 8 |
| CFLAR | CASP8 and FADD like apoptosis regulator |
| RIPK3 | receptor interacting serine/threonine kinase 3 |
| CYBB | cytochrome b-245 beta chain |
| CAMK2A | calcium/calmodulin dependent protein kinase II alpha |
| CAMK2D | calcium/calmodulin dependent protein kinase II delta |
| CAMK2B | calcium/calmodulin dependent protein kinase II beta |
| CAMK2G | calcium/calmodulin dependent protein kinase II gamma |
| SLC25A4 | solute carrier family 25 member 4 |
| SLC25A5 | solute carrier family 25 member 5 |
| SLC25A6 | solute carrier family 25 member 6 |
| SLC25A31 | solute carrier family 25 member 31 |
| PPID | peptidylprolyl isomerase D |
| VDAC1 | voltage dependent anion channel 1 |
| VDAC2 | voltage dependent anion channel 2 |
| VDAC3 | voltage dependent anion channel 3 |
| GLUD2 | glutamate dehydrogenase 2 |
| GLUD1 | glutamate dehydrogenase 1 |
| GLUL | glutamate-ammonia ligase |
| PYGL | glycogen phosphorylase L |
| PYGM | glycogen phosphorylase, muscle associated |
| PYGB | glycogen phosphorylase B |
| MAPK8 | mitogen-activated protein kinase 8 |
| MAPK10 | mitogen-activated protein kinase 10 |
| MAPK9 | mitogen-activated protein kinase 9 |
| FTH1 | ferritin heavy chain 1 |
| FTL | ferritin light chain |
| PLA2G4E | phospholipase A2 group IVE |
| PLA2G4A | phospholipase A2 group IVA |
| JMJD7-PLA2G4B | JMJD7-PLA2G4B readthrough |
| PLA2G4B | phospholipase A2 group IVB |
| PLA2G4C | phospholipase A2 group IVC |
| PLA2G4D | phospholipase A2 group IVD |
| PLA2G4F | phospholipase A2 group IVF |
| ALOX15 | arachidonate 15-lipoxygenase |
| CAPN1 | calpain 1 |
| CAPN2 | calpain 2 |
| SMPD1 | sphingomyelin phosphodiesterase 1 |
| MLKL | mixed lineage kinase domain like pseudokinase |
| PGAM5 | PGAM family member 5, mitochondrial serine/threonine protein phosphatase |
| DNM1L | dynamin 1 like |
| NLRP3 | NLR family pyrin domain containing 3 |
| PYCARD | PYD and CARD domain containing |
| CASP1 | caspase 1 |
| IL1B | interleukin 1 beta |
| CHMP2A | charged multivesicular body protein 2A |
| CHMP2B | charged multivesicular body protein 2B |
| CHMP3 | charged multivesicular body protein 3 |
| RNF103-CHMP3 | RNF103-CHMP3 readthrough |
| CHMP4B | charged multivesicular body protein 4B |
| CHMP4A | charged multivesicular body protein 4A |
| CHMP4C | charged multivesicular body protein 4C |
| CHMP6 | charged multivesicular body protein 6 |
| VPS4B | vacuolar protein sorting 4 homolog B |
| VPS4A | vacuolar protein sorting 4 homolog A |
| CHMP1B | charged multivesicular body protein 1B |
| CHMP1A | charged multivesicular body protein 1A |
| CHMP5 | charged multivesicular body protein 5 |
| CHMP7 | charged multivesicular body protein 7 |
| TRPM7 | transient receptor potential cation channel subfamily M member 7 |
| IL1A | interleukin 1 alpha |
| IL33 | interleukin 33 |
| HMGB1 | high mobility group box 1 |
| TNFSF10 | TNF superfamily member 10 |
| TNFRSF10A | TNF receptor superfamily member 10a |
| TNFRSF10B | TNF receptor superfamily member 10b |
| FASLG | Fas ligand |
| FAS | Fas cell surface death receptor |
| FAF1 | Fas associated factor 1 |
| IFNA1 | interferon alpha 1 |
| IFNA2 | interferon alpha 2 |
| IFNA4 | interferon alpha 4 |
| IFNA5 | interferon alpha 5 |
| IFNA6 | interferon alpha 6 |
| IFNA7 | interferon alpha 7 |
| IFNA8 | interferon alpha 8 |
| IFNA10 | interferon alpha 10 |
| IFNA13 | interferon alpha 13 |
| IFNA14 | interferon alpha 14 |
| IFNA16 | interferon alpha 16 |
| IFNA17 | interferon alpha 17 |
| IFNA21 | interferon alpha 21 |
| IFNB1 | interferon beta 1 |
| IFNG | interferon gamma |
| IFNAR1 | interferon alpha and beta receptor subunit 1 |
| IFNAR2 | interferon alpha and beta receptor subunit 2 |
| IFNGR1 | interferon gamma receptor 1 |
| IFNGR2 | interferon gamma receptor 2 |
| JAK1 | Janus kinase 1 |
| JAK2 | Janus kinase 2 |
| JAK3 | Janus kinase 3 |
| TYK2 | tyrosine kinase 2 |
| STAT1 | signal transducer and activator of transcription 1 |
| STAT2 | signal transducer and activator of transcription 2 |
| STAT3 | signal transducer and activator of transcription 3 |
| STAT4 | signal transducer and activator of transcription 4 |
| STAT5A | signal transducer and activator of transcription 5A |
| STAT5B | signal transducer and activator of transcription 5B |
| STAT6 | signal transducer and activator of transcription 6 |
| IRF9 | interferon regulatory factor 9 |
| EIF2AK2 | eukaryotic translation initiation factor 2 alpha kinase 2 |
| TLR4 | toll like receptor 4 |
| TICAM2 | toll like receptor adaptor molecule 2 |
| TICAM1 | toll like receptor adaptor molecule 1 |
| TLR3 | toll like receptor 3 |
| ZBP1 | Z-DNA binding protein 1 |
| USP21 | ubiquitin specific peptidase 21 |
| SQSTM1 | sequestosome 1 |
| HSP90AA1 | heat shock protein 90 alpha family class A member 1 |
| HSP90AB1 | heat shock protein 90 alpha family class B member 1 |
| TNFAIP3 | TNF alpha induced protein 3 |
| PARP1 | poly(ADP-ribose) polymerase 1 |
| BID | BH3 interacting domain death agonist |
| BAX | BCL2 associated X, apoptosis regulator |
| AIFM1 | apoptosis inducing factor mitochondria associated 1 |
| H2AFX | H2A histone family, member X |
| HIST2H2AC | histone cluster 1 H2A family member C |
| HIST1H2AH | histone cluster 1 H2A family member H |
| HIST1H2AA | histone cluster 1 H2A family member A |
| HIST3H2A | histone cluster 3 H2A family member A |
| H2AFB3 | H2A histone family, member B3 |
| HIST1H2AE | histone cluster 1 H2A family member E |
| HIST1H2AB | histone cluster 1 H2A family member B |
| H2AFY2 | H2A histone family, member Y2 |
| H2AFY | H2A histone family, member Y |
| HIST2H2AA4 | histone cluster 2 H2A family member A4 |
| H2AFJ | H2A histone family, member J |
| H2AFB1 | H2A histone family, member B1 |
| HIST1H2AM | histone cluster 1 H2A family member M |
| HIST2H2AA3 | histone cluster 2 H2A family member A3 |
| HIST1H2AG | histone cluster 1 H2A family member G |
| HIST2H2AB | histone cluster 2 H2A family member B |
| H2AFV | H2A histone family, member V |
| HIST1H2AD | histone cluster 1 H2A family member D |
| H2AFZ | H2A histone family, member Z |
| HIST1H2AK | histone cluster 1 H2A family member K |
| HIST1H2AC | histone cluster 1 H2A family member C |
| HIST1H2AI | histone cluster 1 H2A family member I |
| HIST1H2AJ | histone cluster 1 H2A family member J |
| HIST1H2AL | histone cluster 1 H2A family member L |
| H2AFB2 | H2A histone family, member B2 |
| PPIA | peptidylprolyl isomerase A |
| BCL2 | BCL2 apoptosis regulator |
