# Supplementary material for: Establishment of a Novel Prognostic Prediction Model for Gastric Cancer Based on Necroptosis-Related Genes
Source: Pathol Oncol Res. 2022 Sep 15;28:1610641. doi: 10.3389/pore.2022.1610641 (PMC9519854; doi:10.3389/pore.2022.1610641)
Supplement: Supplementary file 2 [file DataSheet1.docx]

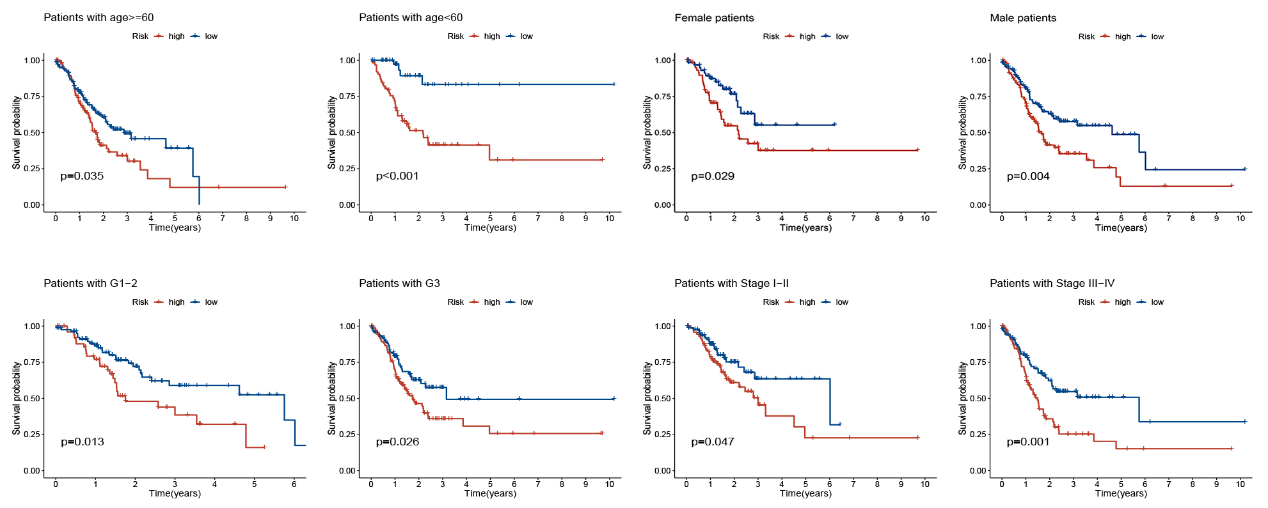


**Supplementary Figure 1** The overall survival rate of patients in two risk groups between distinct clinical variable subgroups.
